# Generalized teleparallel de Sitter geometries

**DOI:** 10.1140/epjc/s10052-023-12150-1

**Published:** 2023-10-30

**Authors:** A. A. Coley, A. Landry, R. J. van den Hoogen, D. D. McNutt

**Affiliations:** 1 Department of Mathematics and Statistics, Dalhousie University, Halifax, NS B3H 3J5 Canada; 2 Department of Mathematics and Statistics, St. Francis Xavier University, Antigonish, NS B2G 2W5 Canada; 3 Department of Mathematics and Statistics, UiT: The Arctic University of Norway, 9037 Tromso, Norway

## Abstract

Theories of gravity based on teleparallel geometries are characterized by the torsion, which is a function of the coframe, derivatives of the coframe, and a zero curvature and metric compatible spin-connection. The appropriate notion of a symmetry in a teleparallel geometry is that of an affine symmetry. Due to the importance of the de Sitter geometry and Einstein spaces within General Relativity, we shall describe teleparallel de Sitter geometries and discuss their possible generalizations. In particular, we shall analyse a class of Einstein teleparallel geometries which have a 4-dimensional Lie algebra of affine symmetries, and display two one-parameter families of explicit exact solutions.

## Introduction

Teleparallel geometries, which are characterized by the torsion (which is a function of the coframe and its derivatives), and a zero curvature and metric compatible spin-connection, provide an alternative to Riemmannian geometries in which to formulate a theory of gravity. A particular subclass of teleparallel gravitational theories is dynamically equivalent to General Relativity (GR) and is often referred to as the Teleparallel Equivalent to General Relativity (TEGR) [1]. In the *covariant* approach to the more general class of *F*(*T*) teleparallel gravity theories [2], the teleparallel geometry is defined in a gauge invariant manner as a geometry having a spin connection with vanishing curvature. The spin connection can be zero in a very special class of “proper” frames and is non-zero in all other frames [1, 2].

The torsion scalar, *T*, is defined in terms of the torsion tensor

1 $$\begin{aligned} T=\frac{1}{2}T^a_{\mu \nu }S_a^{\mu \nu }, \end{aligned}$$

where the superpotential, $S^a_{~\mu \nu }$, is constructed from the torsion tensor by

2 $$\begin{aligned} S_a^{\mu \nu }=\frac{1}{2}\left( T_a^{\mu \nu }+T^{\nu \mu }_{a} -T^{\mu \nu }_{a}\right) -h_a^{\nu }T^{\phi \mu }_{~~\phi } + h_a^{\mu }T^{\phi \nu }_{~~\phi }. \end{aligned}$$

The variations of the Lagrangian $\frac{h}{2\kappa }F(T)+\mathcal {L}_{Matt}$, which include a non-trivial spin-connection [2, 3], lead to *Lorentz covariant* field equations (FEs) and the resulting theory is locally Lorentz invariant [4]. Treating the spin-connection as an independent field, the Lagrangian can then be written using Lagrange multipliers to impose the two constraints of zero curvature and metric compatibility. Assuming an orthonormal frame, the corresponding variations with respect to (w.r.t.) the Lagrange multipliers lead to the following equations (eqns.)

3 $$\begin{aligned} \omega ^a_{b\mu } = \Lambda ^a_{c}\partial _\mu \Lambda _{b}^{{c}} \quad \ \text{ and }\ \quad \omega _{(ab)\mu } = 0, \end{aligned}$$

where $\Lambda ^a_{b}\in SO(1,3)$ (and $\Lambda _b^{c} \equiv (\Lambda ^{-1})^c_{b}$). Variations of the Lagrangian with respect to the coframe can be decomposed into symmetric and antisymmetric parts:

4a $$\begin{aligned} \kappa \Theta _{(ab)}= & {} F''(T)S_{(ab)}^{\nu } \partial _{\nu } T+F'(T){\tilde{G}}_{ab} + \frac{1}{2}g_{ab}\left( F(T)-TF'(T)\right) , \end{aligned}$$

4b $$\begin{aligned} 0= & {} F''(T)S_{[ab]}^{\nu } \partial _{\nu } T, \end{aligned}$$

where ${\tilde{G}}_{ab}$ is the Einstein tensor calculated explicitly from the metric [2]. Note that any quantity with an overtilde is computed using the Levi-Civita connection in terms of the metric. In the above FEs, $\Theta _{(ab)}$ is the matter energy momentum tensor (and $\Theta _{[ab]}=0$ due to the invariance of the FEs under *SO*(1, 3)).

The determination of new solutions in a given *F*(*T*) theory is vital for investigating the validity of the theory. Furthermore, distinguishing a new solution from a known solution can be difficult from the perspective of coordinates, as this necessitates finding the coordinate transformation that brings one solution to the other. As an alternative, it is possible to use torsion invariants to determine the equivalence of two solutions. The Cartan–Karlhede algorithm is an algorithmic approach to generate a geometrically preferred frame and connection which can characterize a given solution in terms of invariant quantities [5].

The algorithm exploits the canonical form of the *q*-th order torsion tensors (that is the torsion tensor and its *q*-th covariant derivatives) to fix the frame. At the zeroth iteration, the torsion tensor is computed and the Lorentz frame freedom is used to adapt the frame so that the torsion tensor takes on a canonical form. The number of functionally independent components, $t_0$, are recorded and the remaining frame freedom, which does not affect the torsion tensor, is denoted as the 0-th order linear isotropy group, $H_0$.

For the *q*-th iteration, the *q*-th order torsion tensor is computed and the $q-1$ linear isotropy group, $H_{q-1}$ is used to determine a canonical form for the *q*-th torsion tensor and the number of functionally independent components, $t_q$, are recorded along with the *q*-th linear isotropy group, $H_q$. The algorithm stops at iteration $p+1$, when $t_p = t_{p+1}$ and $H_p = H_{p+1}$. The subgroup $H_p$ of the Lorentz group is then called the linear isotropy group of the geometry.

The resulting invariant quantities that characterize the geometry are then $\{ t_q\}_{q=0}^{p+1}$, $\{ H_q \}_{q=0}^{p+1}$ and the set of components $\mathcal {T} = \{ T^a_{~bc}, \nabla _{d_1} T^a_{~bc}, \ldots , \nabla _{d_{p+1}} \ldots \nabla _{d_1} T^a_{~bc} \}$. The set $\mathcal {T}$ are torsion invariants known as *Cartan invariants*. If two solutions share the same discrete sequences, with Cartan invariants $\mathcal {T}$ and $\mathcal {T'}$, respectively, equivalence of the solutions can be proven in a non-algorithmic way by solving the equations arising from equating the components of $\mathcal {T}$ and $\mathcal {T'}$.

In teleparallel geometries, the tetrad (or (co)frame) and the corresponding spin-connection replace the metric as the primary objects of study. Consequently, the appropriate notion of symmetry in a teleparallel geometry is that of an affine symmetry (and not a metric symmetry; i.e, a Killing vector). An affine frame or intrinsic symmetry on the frame bundle is a diffeomorphism from the manifold to itself which is characterized by the existence of a vector field, $\textbf{X}$, satisfying [5, 6]:

5 $$\begin{aligned} \mathcal {L}_{\textbf{X}} \text{ h}_a = \lambda _a^{~b} \,\text{ h}_b \text{ and } \mathcal {L}_{\textbf{X}} \omega ^a_{~bc} = 0, \end{aligned}$$

where $\omega ^a_{~bc}$ denotes the spin-connection relative to the geometrically preferred invariantly defined frame $\text{ h}_a$ determined by the Cartan–Karlhede algorithm and $\lambda _a^{~b}$ is an element of the linear isotropy group determined by the algorithm.

There do not exist any teleparallel geometries admitting a maximal group of affine frame symmetries other than Minkowski space [5, 7]. If a four-dimensional teleparallel geometry has a non-zero torsion, then the maximum dimension of the group of affine symmetries is at most seven [5]. Therefore, in analogy with de Sitter geometries in GR, we define the teleparallel de Sitter (TdS) geometry as that nontrivial teleparallel geometry which has a seven dimensional group of symmetries that is also a subgroup of the group of the Killing symmetries of the de Sitter metric [8].

Due to the importance of the de Sitter geometry within GR, we shall study TdS geometries and their generalizations in this paper. After a brief introduction to the TdS geometry, we discuss generalizations of this geometry mathematically and describe some physical applications, with particular emphasis on possible analogues of Einstein spaces. In particular, we shall discuss a class of Einstein teleparallel geometries which have a 4-dimensional Lie algebra of affine symmetries (in the case where the Einstein space parameter $\lambda$ is equal to zero). We display the governing symmetric FEs explicitly in the first of the two solutions of the antisymmetric FEs. We shall investigate power law solutions and display two one-parameter families of explicit solutions (where we can implicitly find the form of *F*(*T*) for each parameter value).

## Teleparallel “de Sitter” (TdS)

We will work in a coframe in which the tangent space metric has the form $g_{ab}= \eta _{ab} = \text{ Diag }[-1,1,1,1]$, which still allows a *O*(1, 3) subgroup of $GL(4,\mathbb {R})$ of residual gauge transformations which leaves the metric $g_{ab}=\eta _{ab}$ invariant. We can then restrict attention to proper ortho-chronous Lorentz subgroups, *SO*(1, 3) or $SO(1,3)^+$. Within this orthonormal gauge choice, the resulting field equations transform homogeneously under the remaining *O*(1, 3) (or *SO*(1, 3) or $SO(1,3)^+$) Lorentz gauge transformations.

Using the Cartan–Karlhede algorithm [5], we can determine the $G_7$ geometries obtained by requiring that the Cartan invariants are all constant. This follows from the formula for the dimension of the affine frame symmetry group, $N = s+4-t_p$, where *s* is the dimension of the linear isotropy group and $t_p$ is the number of functionally independent invariants at the conclusion of the algorithm. If all of the Cartan invariants are constant, then $t_p = 0$ and the dimension of the linear isotropy group is three, yielding a seven-dimensional affine frame symmetry group.

Solving the differential equations arising from the requirement that the only non-trivial components of the Cartan invariants are $T_{abc}$ yields the general case in which $a(t) = A_0 e^{H_0 t}$ is the scale factor, and $H_0$ is a non-zero constant in the teleparallel $k=0$ Robertson–Walker geometry [9]. The special teleparallel geometry case where $H_0=0$, $a(t) = A_0$ a non-zero constant, $k=\pm 1$ and the additional affine frame symmetry $X_7 = \partial _t$ can be considered as the analogue of the Einstein static geometry in GR [8] . In the general case ($H_0\not =0$), the affine frame symmetry is of the form

6 $$\begin{aligned} X_7 = -\frac{1}{H_0} \partial _t + r \partial _r. \end{aligned}$$

The resulting Lie algebra of $${ \{X_I\}_{I=1}^{7}} = \{X_1,X_2,X_3, X_4, X_5,$$$$ X_6, X_7\}$$ is given by

$$\begin{aligned} \begin{array}{lll} {} [X_1,X_5]=X_3, &{} [X_1,X_6]=X_2, &{} [X_1,X_7]=X_1, \\ {} [X_2,X_4]=-X_3, &{} [X_2,X_6]=-X_1, &{} [X_2,X_7]=X_2, \\ {} [X_3,X_4]=X_2, &{} [X_3,X_5]=-X_1, &{} [X_3,X_7]=X_3, \\ {} [X_4,X_5]=-X_6, &{} [X_4,X_6]=X_5, &{} [X_5,X_6]=-X_4. \end{array} \end{aligned}$$

This is a subalgebra of the Lie algebra for the group of metric (Killing) symmetries of de Sitter metric. We therefore define the Teleparallel de Sitter (TdS) geometry as the teleparallel geometry with a $G_7$ Lie group of affine symmetries which is the semi direct product of the one-dimensional subgroup of *O*(1, 4) and the six dimensional Euclidean group *E*(3). Note in this geometry the covariant derivative of the torsion tensor is zero.

### Properties

The diagonal de Sitter co-frame is

7 $$\begin{aligned} h^a= \left[ \begin{array}{c} dt\\ e^{H_0t}\, dr \\ e^{H_0t}\, rd\theta \\ e^{H_0t}\, r\sin (\theta )d\phi \end{array} \right] , \end{aligned}$$

which has the corresponding spin-connection one form

8 $$\begin{aligned} \omega ^a_{~b}= \left[ \begin{array}{cccc} 0 &{} 0&{} 0&{} 0 \\ 0 &{} 0 &{} -d\theta &{} -\sin (\theta ) d\phi \\ 0 &{} d\theta &{} 0 &{} -\cos (\theta ) d\phi \\ 0 &{} \sin (\theta ) d\phi &{} \cos (\theta ) d\phi &{} 0 \end{array} \right] , \end{aligned}$$

or the non-trivial components of the spin-connection are (all indices down)

9 $$\begin{aligned} \omega _{23\theta }=-\omega _{32\theta }= & {} -1, \nonumber \\ \omega _{24\phi }=-\omega _{42\phi }= & {} -\sin (\theta ), \nonumber \\ \omega _{34\phi }=-\omega _{43\phi }= & {} -\cos (\theta ) \end{aligned}$$

(alternatively, one could use the proper de Sitter coframe given in [8]). Using Eqs. (7) and (8) we have that

10 $$\begin{aligned} T^a=H_0 \left[ \begin{array}{c} 0 \\ h^1 \wedge h^2 \\ h^1 \wedge h^3 \\ h^1 \wedge h^4 \end{array} \right] . \end{aligned}$$

The non-trivial components of the torsion tensor are

11 $$\begin{aligned} T^2_{~tr}=-T^2_{~rt}= & {} H_0e^{H_0t}, \nonumber \\ T^3_{~t\theta }=-T^3_{~\theta t}= & {} H_0e^{H_0t} \,r, \nonumber \\ T^4_{~t\phi }=-T^4_{~\phi t}= & {} H_0e^{H_0t} \,r\sin (\theta ). \end{aligned}$$

The torsion tensor is decomposable into three irreducible parts [5]:

12 $$\begin{aligned} T_{abc} = \frac{2}{3}\,\left( t_{abc}-t_{acb}\right) -\frac{1}{3}\,\left( g_{ab}\, V_c-g_{ac}\,V_b\right) +\epsilon _{abcd}\,A^d, \nonumber \\ \end{aligned}$$

where the vector, axial and tensor components are, respectively:

13a $$\begin{aligned} V_a= & {} T^b_{\;\;ba}, \end{aligned}$$

13b $$\begin{aligned} A^a= & {} \frac{1}{6}\,\epsilon ^{abcd}\,T_{bcd}, \end{aligned}$$

13c $$\begin{aligned} t_{(ab)c}= & {} \frac{1}{2}\,\left( T_{abc}+T_{bac}\right) -\frac{1}{6}\,\left( g_ {ca}\,V_b+g_{cb}\,V_a\right) +\frac{1}{3}\,g_{ab}\,V_c.\nonumber \\ \end{aligned}$$

The dual superpotential two form ${}^*S_{a}$ is

14 $$\begin{aligned} {}^*S_a = \left[ \begin{array}{c} 0 \\ -2 H_0e^{2H_0t}\, r^2\sin (\theta )d\theta \wedge d\phi \\ 2 H_0e^{2H_0t}\, r\sin (\theta ) dr \wedge d\phi \\ -2 H_0e^{2H_0t}\, r dr \wedge d\theta \end{array} \right] . \end{aligned}$$

The non-trivial components of ${}^*S_{a}$ are

15 $$\begin{aligned} {}^*S^2_{~\theta \phi }=-{}^*S^2_{~\phi \theta }= & {} -2 H_0 e^{2H_0t}\,r^2\sin (\theta ), \nonumber \\ {}^*S^3_{~r\phi }=-{}^*S^3_{~\phi r}= & {} 2 H_0 e^{2H_0t}\,r\sin (\theta ), \nonumber \\ {}^*S^4_{~r\theta }=-{}^*S^4_{~\theta r}= & {} -2 H_0 e^{2H_0t}\,r, \end{aligned}$$

or the non-dual non-trivial components are

16 $$\begin{aligned} S_{212}=-S_{221}= & {} -2H_0, \nonumber \\ S_{313}=-S_{331}= & {} -2H_0, \nonumber \\ S_{414}=-S_{441}= & {} -2H_0. \end{aligned}$$

The part of the superpotential that comes into the FEs in terms of coordinates is $S_{(ab)}^{~~\mu }$ and $S_{[ab]}^{~~\mu }$. The non trivial components of $S_{[ab]}^{~~\mu }$ are

17 $$\begin{aligned} S_{[12]}^{~~r} = - S_{[21]}^{~~r}= & {} \frac{H_0}{e^{H_0t}}, \nonumber \\ S_{[13]}^{~~\theta } =-S_{[31]}^{~~\theta }= & {} \frac{H_0}{e^{H_0t}\,r}, \nonumber \\ S_{[14]}^{~~\phi } = -S_{[41]}^{~~\phi }= & {} \frac{H_0}{e^{H_0t}\,r\sin (\theta )}. \end{aligned}$$

The non trivial components of $S_{(ab)}^{~~\mu }$ are

18 $$\begin{aligned}{} & {} S_{(12)}^{~~r} =S_{(21)}^{~~r} = -\frac{H_0}{e^{H_0t}}, \nonumber \\{} & {} S_{(13)}^{~~\theta } =S_{(31)}^{~~\theta } = -\frac{H_0}{e^{H_0t}\,r}, \nonumber \\{} & {} S_{(14)}^{~~\phi } =S_{(41)}^{~~\phi } = -\frac{H_0}{e^{H_0t}\,r\sin (\theta )}, \nonumber \\{} & {} S_{22}^{~~t}=S_{33}^{~~t}=S_{44}^{~~t} = -2H_0. \end{aligned}$$

The torsion scalar is $T=6H_0^{\,2}$, the axial part of the torsion scalar is zero, and the magnitude of the vector part is $-9H_0^2$ ($k=0, T=6H_0^{\,2}$; for reference, the vector part is $V^2=-9H_0^{\,2}$ and the axial part $A^2=0$).

Assuming a comoving perfect fluid as the matter source, the FEs in the TdS geometry reduce to

19 $$\begin{aligned} \kappa \,\rho = -\kappa \,P = -\frac{1}{2}F(T_0) + 6F'(T_0)\,H_0^{\,2}, \end{aligned}$$

where necessarily $\rho$ and *P* are constants. The equations formally reduce to their GR counterparts only when $F(T)=T$. We note that the effective equation of state

20 $$\begin{aligned} \omega _{eff}=\frac{P}{\rho }=-1, \end{aligned}$$

is the same as its GR counterpart; however, the effective cosmological constant $\Lambda _{eff}\equiv \kappa \rho$ depends in principle on the two parameters $F(T_0)$ and $F'(T_0)$. The energy-momentum conservation eqn.

21 $$\begin{aligned} {\dot{\rho }} + 3\frac{\dot{a}}{a}(\rho + P) = 0, \end{aligned}$$

is identically satisfied.

## Applications

If $T = \text {const.}$, then the FEs for *F*(*T*) teleparallel gravity are equivalent to a rescaled version of TEGR (which looks like GR with a cosmological constant and a rescaled coupling constant) [2]. In the case of TEGR, where $F(T)=T$, Eq. (4b) vanishes. For $F(T) \ne T$, the variation of the gravitational Lagrangian by the flat spin-connection is equivalent to the antisymmetric part of the FEs in Eq. (4b) [2]. In addition, since the canonical energy momentum is symmetric, $\Theta _{[ab]} = 0,$ then the antisymmetric part of the FEs (4b) limit the possible solutions of spacetimes.

Cosmological models in flat ($k = 0$) TRW models have been studied in [10, 11] (also see references within). For example, simple power-law scale factor solutions in specific *F*(*T*) models (using a priori ansatz such as a polynomial functions) have been investigated. Futhermore, the late time behaviour of the usual de Sitter spacetime, and in particular its stability (as a fixed point), has been studied in [10] (see also [12]).

### Stability

Let us simply consider the linear perturbations of the scalar quantities $T,\,\rho ,\,P$ in the TdS solution:

22a $$\begin{aligned} T= & {} 6\,H_0^{\,2} + T_1, \end{aligned}$$

22b $$\begin{aligned} \kappa \rho= & {} - \frac{F(T_0)}{2} + 6F'(T_0)\,H_0^{\,2} + \kappa \rho _1, \end{aligned}$$

22c $$\begin{aligned} \kappa P= & {} - \left( -\frac{F(T_0)}{2} + 6F'(T_0)\,H_0^{\,2}\right) + \beta \,\kappa \rho _1, \end{aligned}$$

where we have used the zeroth order TdS expressions above with $T_0 \equiv 6H_0^{\,2}$, and we assume the parameter $\beta \ge -\frac{1}{3}$ from the energy conditions.

To first order the antisymmetric FEs yield

23 $$\begin{aligned} S_{0~[ab]}^{~~\gamma } T_{1,\gamma } = 0, \end{aligned}$$

where the non trivial zeroth order components of $S_{[ab]}^{~~\mu }$ are given (by Eqs. (22a) to (22c)) above, so that trivially $T_{1,\gamma } = 0$ and hence $T_1 = T_1(t)$.

The FEs and the conservation law then imply that

24a $$\begin{aligned} \left[ -\frac{1}{2}F'(T_0) + 6F''(T_0) H_0^{\,2}\right] \,T_1= & {} \kappa \rho _1, \end{aligned}$$

24b $$\begin{aligned} - 3H_0(1 + \beta ) \rho _1= & {} \dot{\rho _1}. \end{aligned}$$

We immediate have that $\rho _1 = \rho _1(t)$, so that from the conservation eqn. $\rho _1 \rightarrow 0$ to the future (since $H_0 > 0$), whence (except in the degenerate case in which the term in square brackets above might be zero), $T_1 \rightarrow 0$, and hence $T \rightarrow T_0$, a constant, so that the resulting geometry is formally equivalent to that of GR and the corresponding stability results follow.

### The function *F*(*T*)

A number of particular examples of *F*(*T*) theories studied in the literature include polynomial functions in *T*, and especially quadratic $T^2$ theory [10, 11]. Recently, it has been shown that the theory [13]

25 $$\begin{aligned} F(T) = -\Lambda +T+\gamma \,T^2, \end{aligned}$$

can alleviate a variety of cosmological tensions. It is also of interest to study the theory with

26 $$\begin{aligned} F(T) = T + \gamma T^{\tilde{\beta }}, \end{aligned}$$

where $\tilde{\beta }>0$ ( where $\tilde{\beta }$ is potentially a positive integer greater than 2 for sufficient differentiability), or an exponential function

27 $$\begin{aligned} F(T) = F(0)\,\left[ \exp \left( 2\gamma \,T\right) -1\right] \sim F(0)\,\left[ T + \gamma \,T^2+...\right] \nonumber \\ \end{aligned}$$

for small *T*.

## Analogues of Einstein spaces

In Riemannian geometries the subclass of Einstein spaces with ${ \tilde{R}_{ab}} = \lambda g_{ab}$, and which contain the de Sitter geometry as a special case, are of interest [14]. We note that for Einstein spaces, in which the algebraic structure of the Ricci tensor ${ \tilde{R}_{ab}}$ is special, the frame components of the Ricci tensor ${ \tilde{R}_{ab}}$ are constants and its covariant derivative is zero.

It may be of interest to study analogous particular teleparallel geometries with special properties (but non-constant *T*) which include the TdS geometry. Possible examples might include teleparallel geometries with special algebraic, topological or symmetry properties. Such examples often lead to $\tilde{R}_{ab} = \lambda g_{ab}$ and hence to a subcase of the usual Einstein spaces (which implies the energy momentum tensor $\Theta _{ab} = \lambda g_{ab}$).

### Algebraic

Examples include:

1. The components of the torsion tensor are constant or its covariant derivative is zero.
2. The algebraic properties of some tensor constructed from the torsion tensor are special.

In addition, the torsion tensor can be invariantly decomposed algebraically into its vectorial part $V_a$ (Eq. (13a)), axial part $A_a$ (Eq. (13b)) and tensorial part $T_{abc}$ (Eq. (13c)). Special invariantly defined subcases occur when the axial and tensorial parts are zero (or tensorial and vector parts are zero). For example, the TdS geometry has vanishing axial part zero [6].

### Topological

Special classes of spacetimes have topological properties such as product (or warped product) structures, and hence decomposable and reducible structures [15, 16]. Unlike the usual Riemannian case, it is the frame and spin-connection (and/or the torsion tensor) that should have these splitting properties (rather than the curvature and metric).

In the Riemannian case, the de Rham decomposition theorem states that if the holonomy group of a simply-connected Riemannian manifold preserves a proper subspace of the tangent space (i.e., is reducible), then the tangent space is decomposable (into holonomy invariant subspaces) and the manifold is (locally) isometric to a product manifold (i.e., a Riemannian manifold is locally a product of Riemannian manifolds with irreducible holonomy algebras). The Lorentzian case was studied in [17], utilizing Wu’s theorem [18, 19], that asserts that every simply-connected, complete semi Riemannian manifold is isometric to a product of simply-connected, complete semi-Riemannian manifolds, of which one can be flat and all others are indecomposable or “weakly-irreducible” (i.e., with no nondegenerate invariant subspace under holonomy representation).

### Symmetries

The teleparallel de Sitter (TdS) geometry has a $G_7$ Lie group of affine symmetries which is the semi direct product of a one-dimensional subgroup *O*(1, 4) and a six dimensional Euclidean group *E*(3). We could define an appropriate generalization of TdS by considering spaces with a subalgebra of affine symmetries. For example, if we consider the subalgebra generated by the 3 rotational affine symmetries $X_4 - X_6$ (spherical symmetry) and the special affine symmetry $X_7= -\frac{1}{H_0} \partial _t + r \partial _r$ (Eq. (6)), we obtain a subclass of geometries with a 4-dimensional Lie subgroup as its affine symmetry group, which we shall define as Einstein Teleparallel (ET) geometries. The TdS geometry is then a special case in which there are an additional 3 affine symmetries ($X_1 - X_3$) [6, 9].

## Einstein teleparallel geometries

Relative to the representation for the isotropy group used in [6], we can determine the most general frame and spin-connection with the three spherically symmetric affine frame symmetry generators $X_4 - X_6$ along with the fourth additional affine symmetry $X_7$ defined above (see [6] and also the case $\lambda =0$ in [9]). We can also choose a new coordinate system to “diagonalize” the frame. The resulting veilbein is:

28 $$\begin{aligned} h^a_{~\mu } = \left[ \begin{array}{cccc} A_1(t,r) &{} 0 &{} 0 &{} 0 \\ 0 &{} A_2(t,r) &{} 0 &{} 0 \\ 0 &{} 0 &{} A_3(t,r) &{} 0 \\ 0 &{} 0 &{} 0 &{} A_3(t,r) \sin (\theta ) \end{array}\right] . \end{aligned}$$

With this choice of invariant symmetry frame, we can now obtain the most general metric compatible connection [6]:

29 $$\begin{aligned}&\omega _{341} = X_1(t,r),&\omega _{342}&= X_2(t,r), \nonumber \\&\omega _{233} = \omega _{244} = X_3(t,r),&\omega _{234}&= -\omega _{243} = X_4(t,r), \nonumber \\&\omega _{121} = X_5(t,r),&\omega _{122}&= X_6(t,r), \nonumber \\&\omega _{133} = \omega _{144} = X_7(t,r),&\omega _{134}&= -\omega _{143} = X_8(t,r), \nonumber \\&\omega _{344} = - \frac{\cos (\theta )}{A_3 \sin (\theta )}. \end{aligned}$$

Finally, to determine the most general connection for a teleparallel geometry we must impose the flatness condition. The resulting equations can be solved so that any spherically symmetric teleparallel geometry is defined by (the three arbitrary functions in the veilbein (28) along with) the following spin-connection components [9]:

30 $$\begin{aligned} X_1&= -\frac{\partial _t \chi }{A_1}, \quad \quad \quad \quad \quad&X_2&= -\frac{\partial _r \chi }{A_2}, \nonumber \\ X_3&= \frac{\cosh (\psi )\cos (\chi )}{A_3}, \,\quad&X_4&= \frac{\cosh (\psi )\sin (\chi )}{A_3}, \nonumber \\ X_5&= -\frac{\partial _t \psi }{A_1}, \quad \quad \quad \quad \quad&X_6&= -\frac{\partial _r \psi }{A_2}, \nonumber \\ X_7&= \frac{\sinh (\psi ) \cos (\chi )}{A_3}, \,\quad&X_8&= \frac{\sinh (\psi ) \sin (\chi )}{A_3}, \end{aligned}$$

where $\chi$ and $\psi$ are arbitrary functions of the coordinates *t* and *r*.

Thus, to determine the conditions of the inclusion of the new symmetry generator, we need only examine the conditions $\mathcal {L}_{\textbf{X}_7} \text{ h}^a = 0$, from which we obtain:

31 $$\begin{aligned} A_1 (t,r) = f_1(z),\quad \quad A_2(t,r) = { \frac{f_2(z)}{r} }, \quad \quad A_3(t,r) = f_3(z),\nonumber \\ \end{aligned}$$

where $z \equiv r e^{-C_0 t}$ and $C_0 \equiv -H_0$ is a non-zero constant. The Lie derivatives of the metric *g* and the spin-connection, respectively, yield $\mathcal {L}_{\textbf{X}_7}\,g = 0$ and $\mathcal {L}_{\textbf{X}_7} \omega ^a_{~bc}= 0$, as desired. It therefore follows that the arbitrary functions in the spin-connection’s components must be functions of the form:

32 $$\begin{aligned} \chi (t,r) = \chi (z),\quad \quad \quad \quad \psi (t,r) = \psi (z). \end{aligned}$$

For general situations in which the parameter $\lambda \ne 0$, we have that the torsion scalar $T=T(z,r)$ will also contain terms in $r^\lambda$ as studied in [9] for $T(z,r)=\frac{T_0(z)}{r^{2\lambda }}$. From the FEs we obtain the following equation in term of $r^{-2\lambda }\,F'(T)$:

33 $$\begin{aligned} \kappa \,\left( P + \rho \right)= & {} 2\,\left[ -g_4\,\left[ \frac{\left( h_2 +2\,h_3\right) \,T_0'(z)+ \left( 2\,m_3\right) \,T_0(z)}{h_4\,T_0'(z)+m_4\,T_0(z)}\right] \right. \nonumber \\{} & {} \left. +\left( g_2+g_3\right) \right] \,r^{-2\lambda }\,F'(T), \nonumber \\\equiv & {} 2\,H(z)\,r^{-2\lambda }\,F'(T). \end{aligned}$$

We note that in general with $\lambda \ne 0$, $H(z) \ne 0$ [9].

For $\lambda = 0$, as considered here, we obtain that $T(z,r)=T(z)$. In general, without substituting in the solutions of the antisymmetric FEs solutions (and the symmetric FEs), we have that $H(z) \ne 0$. However, we will see below that, using the solution to the antisymmetric FEs, we find that $H(z)=0$. This then implies that $\rho + P= 0$. This is consistent with the fact that this is intended as an anologue of an Einstein space.

### Antisymmetric FEs and solutions

For $\lambda = 0$, the antisymmetric FEs become (when the torsion scalar $T(z)\ne \text {const}$ and *F*(*T*) is not linear in *T*):

34a $$\begin{aligned}&0 = \sin \,\chi (z)\,\left[ C_0\,f_2(z)\,\sinh \psi (z) + f_1(z)\,\cosh \psi (z)\right] , \end{aligned}$$

34b $$\begin{aligned}&0 = \cos \,\chi (z)\,\left[ C_0\,f_2(z)\,\cosh \psi (z) + f_1(z)\,\sinh \psi (z)\right] . \end{aligned}$$

There are two solutions:

A: $$\cos \,\chi (z)=0$$: $$\sin \,\chi (z)=\delta =\pm 1$$, $$\chi = \left( k+\frac{1}{2}\right) \pi $$ and $$\tanh \psi (z)=-\frac{f_1(z)}{C_0\,f_2(z)}$$.
B: $$\sin \,\chi (z)=0$$: $$\cos \,\chi (z)=\delta =\pm 1$$, $$\chi = k\pi $$ and $$\tanh \psi (z)=-\frac{C_0\,f_2(z)}{f_1(z)}$$.

### General form of symmetric FEs

The general symmetric FEs can be expressed in the following form:

35a $$\begin{aligned} k_1\,\partial _z\left( \ln (F'(T))\right)= & {} g_1, \end{aligned}$$

35b $$\begin{aligned} \kappa \,P -\frac{F(T)}{2}= & {} 2\,F'(T)\,\left[ -k_2\,\partial _z\left( \ln (F'(T))\right) +g_2\right] , \end{aligned}$$

35c $$\begin{aligned} \kappa \,\rho +\frac{F(T)}{2}= & {} 2\,F'(T)\,\left[ -k_3\,\partial _z\left( \ln (F'(T))\right) +g_3\right] , \end{aligned}$$

35d $$\begin{aligned} k_4\,\partial _z\left( \ln (F'(T))\right)= & {} g_4, \end{aligned}$$

where the functions $g_i$ and $k_i$ ($i=1-4$) are explicitly displayed below for solution A (the functions for solution B are displayed in the Appendix). By combining the pair of Eqs. (35b) and (35c) and then Eqs. (35a) and (35d), respectively, we obtain:

36a $$\begin{aligned} \partial _z\left( \ln (F'(T))\right)= & {} \frac{g_1}{k_1} = \frac{g_4}{k_4}, \end{aligned}$$

36b $$\begin{aligned} \kappa \,\left( P+\rho \right)= & {} 2\,F'(T)\left[ -\left( k_2+k_3\right) \,\partial _z\left( \ln (F'(T))\right) + \left( g_2+g_3\right) \right] \nonumber \\{} & {} \equiv F'(T) H(z). \end{aligned}$$

The general torsion scalar expression respecting Eqs. (36a) and (36b) is the following:

37 $$\begin{aligned} T(z)&= \frac{1}{f_1^2\,f_2^2\,f_3^2}\Bigg [-4\,f_1\,f_2\,z\,\cos \chi \Bigg (\left[ \left( f_3\,f_1\right) '+f_3\,f_2\,\psi '\right] \cosh \psi \nonumber \\&\quad +\sinh \psi \left[ \psi '\,f_1\,f_3+C_0\left( f_2\,f_3\right) '\right] \Bigg ) \nonumber \\&\quad +4\,z\,\sin \chi \,\cosh \psi \,\chi '\,f_1^2\,f_2\,f_3\nonumber \\&\quad +4\,C_0\,z\,\sin \chi \,\sinh \psi \,\chi '\,f_1\,f_2^2\,f_3-2\,f_1^2\left( z^2\,f_3'^2+f_2^2\right) \nonumber \\&\quad -4\,z^2\,f_1\,f_3\,f_1'\,f_3' +4\,C_0^2\,z^2\,f_2\,f_3'\left( f_2'\,f_3+\frac{f_2\,f_3'}{2}\right) \Bigg ] . \end{aligned}$$

### Coordinates

There is a class of transformations that maintain the “diagonal” form of the frame in (28). However, in general, this will also lead to a change in the form of the connection and the FEs. Therefore, in order to maintain the comoving nature of the perfect fluid source, we restrict ourselve to transformations of the form $t \rightarrow f(t)$ and $r \rightarrow g(r)$ (i.e., redefining the *r* and *t* coordinates). This may simplify the eqns. further. Even then, the explicit forms for the symmetry vectors and the similarity variable *z* (for example) does change. We shall not change coordinates here.

However, since the static case corresponds formally to $C_0=0$, we assume explicitly that $C_0^2 \ne 0$ here. In this case we can then effect a simple time change to set $C_0^2 =1$, which we shall do hereafter. This has the effect of changing the metric, $-dt^2 \rightarrow - \frac{1}{H_0^2}dt^2$. In the exact TdS solutions $C_0^2 =1$ and the metric changes accordingly.

### Solution A

The symmetric FE components (see Eqs. (35a) to (35d)) for solution A (with $C_0^2 = 1$) are explicitly given by (the symmetric FEs components for solution B are displayed in the Appendix):

38a $$\begin{aligned} g_1 =&\Bigg [-z^2\,f_1\,f_2\,f_3\,f_3''\left( f_1^2+f_2^2\right) -z^2\,f_1^2\,f_2\,f_3^2\,f_1''+z^2\,f_1\,f_2^2\,f_3^2\,f_2''\nonumber \\&+z^2\,f_3'^2\left( -f_1\,f_2^3 + f_1^3\,f_2\right) \nonumber \\&+f_3\,f_3'\,\left( f_2^2+f_1^2\right) \left( z^2\,f_2\,f_1'+f_1\,\left( z^2\,f_2'-z\,f_2\right) \right) \nonumber \\&+f_3^2\,f_1'\,\left( \left( f_1^2-f_2^2\right) \,z^2\,f_2'-z\,f_1^2\,f_2\right) \nonumber \\&+f_1\,f_2^2\,\left( z\,f_3^2\,f_2'-f_1^2\,f_2\right) \Bigg ] , \end{aligned}$$

38b $$\begin{aligned} g_2 =&\frac{1}{f_1^3\,f_2^2\,f_3^2}\Bigg [-z^2\,f_1\,f_2^2\,f_3\,f_3''+z^2\,f_1\,f_3'^2\,\left( f_1^2-f_2^2\right) \nonumber \\&+f_3\,f_3'\Bigg (z^2\,f_1'\left( f_2^2+2\,f_1^2\right) \nonumber \\&-f_1\,f_2\,\left( z^2\,f_2'+z\,f_2\right) \Bigg )\Bigg ] , \end{aligned}$$

38c $$\begin{aligned} g_3 =&\frac{1}{f_1^2\,f_2^3\,f_3^2}\Bigg [-z^2\,f_3''\,f_3\,f_1^2\,f_2+f_2\,f_3'^2\,z^2\left( f_2^2-f_1^2\right) \nonumber \\&-f_3'\Bigg (-z^2\,f_3\,f_2'\left( 2\,f_2^2+f_1^2\right) \nonumber \\&+f_1\,f_2\left( z^2\,f_3\,f_1'+zf_1\,f_3\right) \Bigg )\Bigg ] , \end{aligned}$$

38d $$\begin{aligned} \nonumber \\ g_4 =&\Bigg [\ln \left( \frac{f_1\,f_2}{z\,f_3'}\right) \Bigg ]' , \end{aligned}$$

39a $$\begin{aligned} k_1 =&\left( z^2\,f_1\,f_2\,f_3\right) \,\Bigg [f_3'\left( f_1^2+f_2^2\right) +f_3\,\left( f_1\,f_1'-f_2\,f_2'\right) \Bigg ] , \end{aligned}$$

39b $$\begin{aligned} k_2 =&\frac{z^2\,f_3'}{f_1^2\,f_3} , \end{aligned}$$

39c $$\begin{aligned} k_3 =&\frac{z^2\,f_3'}{f_2^2\,f_3} , \end{aligned}$$

39d $$\begin{aligned} k_4 =&1 . \end{aligned}$$

We simplify the symmetric FEs for solution A into Eq. (36a), to obtain:

40 $$\begin{aligned}&\Bigg [-z^2\,f_1\,f_2\,f_3\,f_3''\left( f_1^2+f_2^2\right) -z^2\,f_1^2\,f_2\,f_3^2\,f_1''\nonumber \\&\qquad +z^2\,f_1\,f_2^2\,f_3^2\,f_2''+z^2\,f_3'^2\left( -f_1\,f_2^3 +f_1^3\,f_2\right) \nonumber \\&\qquad +f_3\,f_3'\,\left( f_2^2+f_1^2\right) \left( z^2\,f_2\,f_1'+f_1\,\left( z^2\,f_2'-z\,f_2\right) \right) \nonumber \\&\qquad +f_3^2\,f_1'\,\left( \left( f_1^2-f_2^2\right) \,z^2\,f_2'-z\,f_1^2\,f_2\right) \nonumber \\&\qquad +f_1\,f_2^2\,\left( z\,f_3^2\,f_2'-f_1^2\,f_2\right) \Bigg ]\Bigg [\left( z^2\,f_1\,f_2\,f_3\right) \nonumber \\&\qquad \times \Bigg [f_3'\left( f_1^2+f_2^2\right) +f_3\,\left( f_1\,f_1'-f_2\,f_2'\right) \Bigg ]\Bigg ]^{-1} \nonumber \\&\quad =-\left( \ln \left( \frac{z\,f_3'}{f_1\,f_2}\right) \right) ' . \end{aligned}$$

Using the eqns. above and the symmetric FE, we find that the right-hand side of Eq. (36b) vanishes, thus giving $P+\rho = 0$. So, for $\lambda =0$, we only have $\kappa P(t,r)=- \kappa \rho (t,r) \equiv -\Lambda _0$, regardless of the form of *F*(*T*(*z*)). This is consistent with the fact that this is intended as an anologue of an Einstein space. This means that we have the following relations:

41 $$\begin{aligned} \left( k_2+k_3\right) \,g_4= & {} \left( g_2+g_3\right) , \nonumber \\ -\left( \ln \left( \frac{z\,f_3'}{f_1\,f_2}\right) \right) '= & {} g_4 =\frac{g_2+g_3}{k_2+k_3}. \end{aligned}$$

By putting the Eqs. (40) and (41) together, we arrive at the following superrelation:

42 $$\begin{aligned} -\left( \ln \left( \frac{z\,f_3'}{f_1\,f_2}\right) \right) ' = g_4 =\frac{g_2+g_3}{k_2+k_3}=\frac{g_1}{k_1}. \end{aligned}$$

The torsion scalar expression for solution A simplifies and is given explicitly by:

43 $$\begin{aligned} T(z) =&\frac{2\,z^2\,f_3'^2(z)\left( f_2^2(z)-f_1^2(z)\right) +4z^2\,f_3(z)\,f_3'(z)\left( f_2(z)\,f_2'(z)-f_1(z)\,f_1'(z)\right) -2\,f_1^2(z)\,f_2^2(z)}{f_1^2(z)\,f_2^2(z)\,f_3^2(z)} . \end{aligned}$$

### Explicit equations: summary

First we satisfied the Antisymmetric FEs (Eqs. (34a) and (34b)) leading to solutions A and B. Then we set $C_0^2 =1$ in all of our physical quantities. For the symmetric FEs, we have Eqs. (35a) to (35d) with $g_i$ and $k_i$ ($i=1-4$) given by Eqs. (38a) to (39d), respectively. We restrict to solution A hereafter, whence Eq. (36a) becomes Eq. (40) (see also Eqs. (41) and (42)). After finding $H(z)=0$, Eq. (36b) implies that $\rho +P=0$. Then the torsion scalar *T*(*z*) is given explicitly by Eq. (43). The FE described by Eq. (35d), using $k_4=1$ and the expression above for $g_4$, is replaced by the *first integral*

44 $$\begin{aligned} \frac{dF}{dT}=B_0\left( \frac{f_1\,f_2}{z\,f_3'}\right) , \end{aligned}$$

which is a function of *z*, and where $B_0$ is a constant. The remaining FE described by Eq. (35c) yields an eqn. for *F*(*T*(*z*)) in terms of *z*. We have that $\kappa \rho =- \kappa P=\Lambda _0$, where we have used solution A and set $C_0^2 =1$. In principle, the three remaining FEs, Eqs. (35c), (40) and (44) are then 3 ODEs for the 3 functions $f_i(z)$ for a given *F*(*T*(*z*)).

For example, for the quadratic function *F*(*T*) explicitly given by Eq. (25), we find that $F^\prime = 1 + 2 \gamma T, F^{\prime \prime } = 2 \gamma$ which, when substituted into Eq. (44), yields

45 $$\begin{aligned} T = -\frac{1}{2 \gamma } + \frac{B_0}{2 \gamma } \left( \frac{f_1\,f_2}{z\,f_3'}\right) , \end{aligned}$$

whence using (43) yields a first order ODE for the $f_i$. Equation (35c) then yields a quadratic eqn. for *T* in terms of functions of *z* (including a term $\frac{dT}{dz}$) which upon using Eq. (43) yields a second order ODE for the $f_i$. Equation (40) constitutes another second order ODE for the $f_i$.

We note again that all potential solutions considered here (other than TdS explicitly) are not analogues of GR solutions and are consequently new.

## Power law solutions

To find solutions to the symmetric part of the FEs describing an Einstein teleparallel geometry, we use the following ansatz:

46 $$\begin{aligned} f_1(z)=a_0\,z^a, \quad \quad f_2(z)=b_0\,z^b, \quad \quad f_3(z)=c_0\,z^c, \end{aligned}$$

where $a_0,\,b_0,\,c_0 \ne 0$. Using the solution A, from Eq. (35c) we obtain:

47 $$\begin{aligned} \Lambda _0=&-\frac{F(T(z))}{2}+2\,F'(T(z))\,\Bigg [\frac{c\,(2b+c)}{a_0^2}\,z^{-2a}-\frac{c\,(2a+c)}{b_0^2}\,z^{-2b}\Bigg ] . \end{aligned}$$

We need the exact expressions for *T*(*z*) and *F*(*T*(*z*)) before simplifying further. Using $g_1=g_4\,k_1$, we obtain from Eq. (40):

48 $$\begin{aligned} 0&= z^{a+b}\Bigg [a_0^2\,c_0^2\,\left( c^2-2a^2+ac\right) \,z^{2a+2c}\nonumber \\&\quad -b_0^2\,c_0^2\,\left( c^2-2b^2+bc\right) \,z^{2b+2c}-a_0^2\,b_0^2\,z^{2a+2b}\Bigg ]. \end{aligned}$$

For the torsion scalar, we obtain:

49 $$\begin{aligned} T(z) =&2\Bigg [\frac{c(2b+c)}{a_0^2}\,z^{-2a}-\frac{c(2a+c)}{b_0^2}\,z^{-2b}-\frac{1}{c_0^2}z^{-2c}\Bigg ] . \end{aligned}$$

The derivative $T'(z)$ is:

50 $$\begin{aligned} T'(z) =&-4\,c\left[ \frac{(2b+c)\,a}{a_0^2}\,z^{-2a-1}-\frac{(2a+c)\,b}{b_0^2}\,z^{-2b-1}-\frac{1}{c_0^2}z^{-2c-1}\right] . \end{aligned}$$

From Eq. (44) we obtain:

51 $$\begin{aligned} \frac{dF(T)}{dT}= & {} \frac{{ B_1}}{c}\,z^{a+b-c}, \end{aligned}$$

where ${ B_1}$ is a constant (the degenerate case $c=0$ is not included). For $c=a+b$ we get exactly the TEGR solution. We will consider $a+b \ne c$ for a non-trivial *F*(*T*) solution.

We multiply by the derivative of the torsion scalar to obtain:

52 $$\begin{aligned} \frac{dF(T(z))}{dz}= & {} \frac{dF(T)}{dT}\,T'(z)\nonumber \\= & {} -4{B_1}\,\left[ \frac{(c+2b)\,a}{a_0^2}\,z^{b-c-a-1}\right. \nonumber \\{} & {} \quad \left. -\frac{(c+2a)\,b}{b_0^2}\,z^{a-c-b-1}-\frac{1}{c_0^2}z^{a+b-3c-1}\right] .\nonumber \\ \end{aligned}$$

From this equation, we may integrate $\frac{dF(T(z))}{dz}$ w.r.t *z* (depending on the power of *z* – some situations may lead to $z^{-1}$ and care should be taken). From Eq. (48), we can solve the symmetric FEs for *a*, *b* and *c* and then verify Eq. (47) by taking into account Eqs. (49) to (52) for each possible situation. In principle, there may be some free parameters remaining.

### The possible solutions

If we consider Eq. (48) only there are, in principle, the following possibilities:

1. $a=b$ (with $a_0^2\,b_0^2 \ne 0$), which is not possible because we need to satisfy:
  53 $$\begin{aligned} 0= \left( c^2-2a^2+ac\right) \,c_0^2\Bigg [a_0^2-b_0^2\Bigg ] \quad \text {and}\quad 0= a_0^2\,b_0^2 . \end{aligned}$$
  This case includes the special case $a=b=c$.
2. $a=c$: which leads to:
  54 $$\begin{aligned} 0&= -\Bigg [c_0^2\,\left( c^2-2b^2+bc\right) +a_0^2\Bigg ]\,b_0^2 , \end{aligned}$$
  which gives the following relation between *a* and *b*:
  55 $$\begin{aligned} (a+2b)(a-b)=-\frac{a_0^2}{c_0^2}, \end{aligned}$$
  where, according to Eq. (55), $-2b< a < +b$ for viable solutions.
3. $b=c$: which leads to
  56 $$\begin{aligned} 0=&\Bigg [c_0^2\,\left( b^2-2a^2+ab\right) -b_0^2\Bigg ]. \end{aligned}$$
  Equation (56) leads to the following relation between *a* and *b*:
  57 $$\begin{aligned} (b+2a)(b-a)=\frac{b_0^2}{c_0^2}, \end{aligned}$$
  where it follows that $-\frac{b}{2}< a < +b$ for viable solutions.

### Case I: $a=c$

For $a=c$ and $\frac{a_0^2}{c_0^2}=-(a+2b)(a-b)$ (Eq. (55)), we then compute Eqs. (49), (51) and (52):

58a $$\begin{aligned} T(z)&= 2\Bigg [(a+2b)(2a-b)\,\frac{z^{-2a}}{a_0^2}-\frac{3a^2}{b_0^2}\,z^{-2b}\Bigg ] , \end{aligned}$$

58b $$\begin{aligned} \frac{dF(T)}{dT}&= F'(T(z)) = \frac{{ B_1}}{a}\,z^{b} , \end{aligned}$$

58c $$\begin{aligned} \frac{dF(T(z))}{dz}&= -4{ B_1}\,\left[ \frac{(a+2b)(2a-b)}{a_0^2}\,z^{b-2a-1}-\frac{3a\,b}{b_0^2}\,z^{-b-1}\right] . \end{aligned}$$

When we attempt to integrate Eq. (58c), we see that there may be special cases ($b=0$ or $a=\frac{b}{2}$) which yield a $z^{-1}$ power term, but in each case the corresponding coefficient is zero and therefore no power of $z^{-1}$ terms ever occurs in Eq. (58c). From this, we can integrate Eq. (58c) giving the relation:

59 $$\begin{aligned} F(T(z))&= -4{ B_1}\,\left[ -\frac{(a+2b)}{a_0^2}\,z^{b-2a}+\frac{3a}{b_0^2}\,z^{-b}\right] +C, \end{aligned}$$

where *C* is a constant. By substituting Eq. (59) into Eq. (47), we find that:

60 $$\begin{aligned} \Lambda _0 +\frac{C}{2}&= 2{ B_1}\,\left[ \frac{(a+2b)}{a_0^2}-\frac{(a+2b)}{a_0^2}\right] \,z^{b-2a}=0. \end{aligned}$$

This means that we have $C=-2\Lambda _0$ in Eq. (59) ($b \ne 0$). This constitutes a one parameter (*b* say) family of solutions. It is possible to locally find the inverse $z=z(T)$ from Eq. (58a) to explicitly find *F*(*T*).

As a simple albeit special example, if $a=\frac{b}{2}$, Eqs. (58a) and (58b) become:

61a $$\begin{aligned} z^{b}&= \sqrt{\frac{3}{2}}\,\frac{b}{b_0\,\sqrt{-T}} , \end{aligned}$$

61b $$\begin{aligned} \frac{dF(T)}{dT}&= \frac{\sqrt{24}\,{ B_1}}{2\,b_0\,\sqrt{-T}}\equiv -\frac{F_1}{2\sqrt{-T}} , \end{aligned}$$

where $F_1$ is a constant. We integrate Eq. (61b) w.r.t. to *T* to obtain:

62 $$\begin{aligned} F(T)&=F_1\,\sqrt{-T}+C, \end{aligned}$$

for $T \le 0$. By substituting Eq. (62) into Eq. (47), we get that:

63 $$\begin{aligned} C&= - \frac{5\,b\,b_0\, F_1}{2\,a_0^2}\,\sqrt{\frac{2}{3}}-2\Lambda _0 \equiv F_0-2\Lambda _0, \end{aligned}$$

where $F_0$ is also a constant.

### Case II: $b=c$

For $b=c$ and $\frac{b_0^2}{c_0^2}=(b+2a)(b-a)$ (Eq. (57)), we must evaluate Eqs. (49), (51) and (52) (for $a \ne 0$):

64a $$\begin{aligned} T(z)&= 2\Bigg [\frac{3b^2}{a_0^2}\,z^{-2a}-\frac{(2b-a)(2a+b)}{b_0^2}\,z^{-2b}\Bigg ] , \end{aligned}$$

64b $$\begin{aligned} \frac{dF(T)}{dT}&= F'(T(z)) = \frac{{ B_1}}{b}\,z^{a} , \end{aligned}$$

64c $$\begin{aligned} \frac{dF(T(z))}{dz}&= -4{ B_1}\,\left[ \frac{3b\,a}{a_0^2}\,z^{-a-1}-\frac{(2b-a)(2a+b)}{b_0^2}\,z^{a-2b-1}\right] . \end{aligned}$$

When we attempt to integrate Eq. (64c), we see that there may be special cases when $a=0$ or $a=2b$ which yield $z^{-1}$ power terms, but in each case the corresponding coefficient is zero and therefore such a power of $z^{-1}$ never occurs in Eq. (64c). We can consequently integrate Eq. (64c), giving the relation:

65 $$\begin{aligned} F(T(z))&= 4{ B_1}\,\left[ \frac{3b}{a_0^2}\,z^{-a}-\frac{(2a+b)}{b_0^2}\,z^{a-2b}\right] +C. \end{aligned}$$

By substituting Eq. (65) into Eq. (47), we find that:

66 $$\begin{aligned} \Lambda _0 +\frac{C}{2}&= -2{ B_1}\,\left[ \frac{(2a+b)}{b_0^2}-\frac{(2a+b)}{b_0^2}\right] \,z^{a-2b} = 0. \end{aligned}$$

This again implies that $C=-2\Lambda _0$ as an integrating constant in Eq. (65). In general, it is possible to locally find the inverse $z=z(T)$ from Eq. (64a) then use it to compute *F*(*T*). Note that from Eqs. (64a) and (65), in the TEGR case we must have $a=0$ (which corresponds to the special case $a+b=c$), where

67 $$\begin{aligned} z^{-2b} =&\frac{3b_0^2}{2\,a_0^2}-\frac{b_0^2}{4b^2}\,T. \end{aligned}$$

## Concluding remarks

Since the de Sitter geometry (and Einstein spaces in more generality) within GR have a number of important mathematical and physical applications, we have studied TdS geometries and their generalizations in theories of gravity based on teleparallel geometries. In particular, we have investigated a class of Einstein teleparallel geometries which have a 4-dimensional Lie algebra of affine symmetries. We solved the resulting antisymmetric FEs and we displayed all of the remaining governing eqns. explictly. We then investigated power law solutions and displayed two one-parameter families (within solution class A) of explicit Einstein teleparallel solutions (where we can implicitly find the form of *F*(*T*) for each parameter value). Very few explicit non-trivial exact solutions are known within teleparallel gravity, and the two one-parameter families of exact power law solutions obtained (being possible analogues of Einstein spaces) may have a number of important applications in cosmology and astrophysics.

## Data Availability

This manuscript has no associated data or the data will not be deposited. [Authors’ comment: There is no data as such; everything is done analytically and displayed].

## Expressions for solution B

The functions in the symmetric FEs (Eqs. (35a) to (35d)) for solution B are explicitly given by:

68 $$\begin{aligned} g_1 =&\Bigg [-z^2\,f_1\,f_2\,f_3\,f_3''\,\left( f_1^2+f_2^2\right) -z^2\,f_1^2\,f_2\,f_3^2\,f_1''\nonumber \\&+ \,z^2\,f_1\,f_2^2\,f_3^2\,f_2''+z^2\,f_3'^2\,\left( -f_1\,f_2^3+f_1^3\,f_2\right) \nonumber \\ {}&+ f_3\,f_3'\,\left( f_1^2+ \,f_2^2\right) \left( z^2\,\left( f_1\,f_2\right) '-z\,f_1\,f_2\right) \nonumber \\&+f_3^2\,f_1'\,\left( z^2\,f_2'\left( f_1^2-f_2^2\right) -z\,f_1^2\,f_2\right) \nonumber \\&+f_1\,f_2^2\,\left( z\,f_3^2\,f_2'-f_1^2\,f_2\right) \Bigg ] , \nonumber \\ g_2 =&\frac{1}{f_1^3\,f_2^2\,f_3^2}\Bigg [-z^2\,f_1\,f_2^2\,f_3\,f_3''\nonumber \\&+z^2\,f_3'^2\left( -f_1\,f_2^2+f_1^3\right) +2\,f_3'{\Bigg (}z^2\,f_3\,f_1'\left( \frac{f_2^2}{2}+f_1^2\right) \nonumber \\&-\frac{f_1\,f_2}{2}\Bigg [z^2\,f_3\,f_2'-\delta \,z\Bigg (\frac{f_1^2-f_2^2}{\sqrt{1-\frac{f_2^2}{f_1^2}}}\Bigg )+z\,f_2\,f_3\Bigg ]{\Bigg )}\nonumber \\&+\delta \,z\,f_1\,f_2\,f_3\,\frac{f_1\,f_1'-f_2\,f_2'}{\sqrt{1-\frac{f_2^2}{f_1^2}}}\Bigg ] ,\nonumber \\ g_3 =&\frac{1}{f_1^2\,f_2^3\,f_3^2}\Bigg [-z^2\,f_1^2\,f_2\,f_3\,f_3''+f_3'^2\,z^2\left( f_2^3-f_1^2\,f_2\right) \nonumber \\&-f_3'{\Bigg [}-z^2\,f_3\,f_2'\left( 2\,f_2^2+f_1^2\right) \nonumber \\&+f_1\,f_2\,\Bigg (z^2\,f_1'\,f_3+\Bigg [\delta \,z\,f_2\,\Bigg (\frac{f_1^2-f_2^2}{f_1\,\sqrt{1-\frac{f_2^2}{f_1^2}}}\Bigg )+z\,f_1\,f_3\Bigg ]\Bigg )\Bigg ]\nonumber \\&-\delta \,z\,f_1\,f_2^2\,f_3\Bigg (\frac{f_1\,f_1'-f_2\,f_2'}{f_1\,\sqrt{1-\frac{f_2^2}{f_1^2}}}\Bigg )\Bigg ], \nonumber \\ g_4 =&\Bigg [\ln \left( \frac{f_1\,f_2}{z\,f_3'}\right) \Bigg ]' , \end{aligned}$$

69 $$\begin{aligned} k_1 =&\left[ z^2\,f_1\,f_2\,f_3\,f_3'\,\left( f_1^2+f_2^2\right) \right. \nonumber \\&\left. +f_1\,f_2\,f_3\left( z^2\,f_1\,f_3\,f_1'-f_2\,\left( z^2\,f_3\,f_2'-\delta \,z\,\frac{f_1^2-f_2^2}{\sqrt{1-\frac{f_2^2}{f_1^2}}} \right) \right) \right] ,\nonumber \\ k_2 =&\, \frac{z}{f_1^2\,f_3}\,\left[ \frac{\delta \,f_2}{\sqrt{1-\frac{f_2^2}{f_1^2}}}+z\,f_3'\right] , \nonumber \\ k_3 =&\, \frac{z}{f_2^2\,f_3}\,\left[ \frac{\delta \,f_2}{\sqrt{1-\frac{f_2^2}{f_1^2}}}+z\,f_3'\right] , \nonumber \\ k_4 =&\, \frac{1}{z\,f_3'} \left[ \frac{\delta \,f_2}{\sqrt{1-\frac{f_2^2}{f_1^2}}} + z\,f_3'\right] . \end{aligned}$$

From Eq. (36a) we explictly get:

70 $$\begin{aligned}&\left[ z^2\,f_1\,f_2\,f_3\,f_3'\,\left( f_1^2+f_2^2\right) +f_1\,f_2\,f_3\left( z^2\,f_1\,f_3\,f_1'-f_2\,\right. \right. \nonumber \\&\left. \left. \qquad \times \left[ z^2\,f_3\,f_2'-\delta \,z\,\frac{f_1^2-f_2^2}{\sqrt{1-\frac{f_2^2}{f_1^2}}}\right] \right) \right] ^{-1}\nonumber \\&\qquad \times \Bigg [-z^2\,f_1\,f_2\,f_3\,f_3''\,\left( f_1^2+f_2^2\right) -z^2\,f_1^2\,f_2\,f_3^2\,f_1''\nonumber \\&\qquad +z^2\,f_1\,f_2^2\,f_3^2\,f_2''+z^2\,f_3'^2\,\left( -f_1\,f_2^3+f_1^3\,f_2\right) \nonumber \\&\qquad + f_3\,f_3'\,\left( f_1^2+f_2^2\right) \left( z^2\,\left( f_1\,f_2\right) '-z\,f_1\,f_2\right) \nonumber \\&\qquad +f_3^2\,f_1'\,\left( z^2\,f_2'\left( f_1^2-f_2^2\right) -z\,f_1^2\,f_2\right) \nonumber \\&\qquad +f_1\,f_2^2\,\left( z\,f_3^2\,f_2'-f_1^2\,f_2\right) \Bigg ] \nonumber \\&\quad =\left[ 1+\frac{\delta \,f_2}{z\,f_3'\,\sqrt{1-\frac{f_2^2}{f_1^2}}}\right] ^{-1}\,\Bigg [\ln \left( \frac{f_1\,f_2}{z\,f_3'}\right) \Bigg ]' . \end{aligned}$$

Using Eqs. (68) and (69) and the symmetric FE, we find that the right-hand side of Eq. (36b) vanishes, thus giving $P+\rho = 0$. So, for $\lambda =0$, we have $P(t,r)=-\rho (t,r) \equiv -\Lambda _0$, regardless of the form of *F*(*T*(*z*)). This is consistent with the fact that this is intended as an anologue of an Einstein space. This means that we have the following relation:

71 $$\begin{aligned}{} & {} \left[ 1+\frac{\delta \,f_2}{z\,f_3'\,\sqrt{1-\frac{f_2^2}{f_1^2}}}\right] ^{-1}\,\left[ \ln \left( \frac{f_1\,f_2}{z\,f_3'}\right) \right] ' \nonumber \\{} & {} \quad = \frac{g_4}{k_4} = \frac{g_2+g_3}{k_2+k_3}. \end{aligned}$$

By putting the Eqs. (70) and (71) together, we arrive at the following superrelation:

72 $$\begin{aligned}{} & {} \left[ 1+\frac{\delta \,f_2}{z\,f_3'\,\sqrt{1-\frac{f_2^2}{f_1^2}}}\right] ^{-1}\,\left[ \ln \left( \frac{f_1\,f_2}{z\,f_3'}\right) \right] ' \nonumber \\{} & {} \quad = \frac{g_4}{k_4} =\frac{g_2+g_3}{k_2+k_3}=\frac{g_1}{k_1}. \end{aligned}$$

The expression for the torsion scalar in the second solution then simplifies further.

## References

[1] R. Aldrovandi, J.G. Pereira, *Teleparallel Gravity, Fundamental Theories of Physics*, vol. 173 (Springer, Dordrecht, 2013)

[2] M. Krssak, R.J. van den Hoogen, J.G. Pereira, C.G. Boehmer, A.A. Coley, Teleparallel theories of gravity: illuminating a fully invariant approach. Class. Quantum Gravity **36**(18), 183001 (2019). arXiv:1810.12932 [gr-qc] 10.1088/1361-6382/ab2e1f

[3] M. Krssak, E.N. Saridakis, The covariant formulation of gravity. Class. Quantum Gravity **33**, 115009 (2016). arXiv:1510.08432 [gr-qc] 10.1088/0264-9381/33/11/115009

[4] M. Krssak, J.G. Pereira, Spin connection and renormalization of teleparallel action. Eur. Phys. J. C **75**, 519 (2015). arXiv:1504.07683 [gr-qc] 10.1140/epjc/s10052-015-3749-2

[5] A.A. Coley, R.J. van den Hoogen, D.D. McNutt, Symmetry and equivalence in teleparallel gravity. J. Math. Phys. **61**, 072503 (2020). arXiv:1911.03893 [gr-qc] 10.1063/5.0003252

[6] D.D. McNutt, A.A. Coley, R.J. van den Hoogen, A frame based approach to computing symmetries with non-trivial isotropy groups. J. Math. Phys. **64**, 032503 (2023). arXiv:2302.11493 [gr-qc]

[7] M. Hohmann, L. Järv, M. Krssak, C. Pfeifer, Modified teleparallel theories of gravity in symmetric spacetimes. Phys. Rev. D **100**, 084002 (2019). arXiv:1901.05472 [gr-qc] 10.1103/PhysRevD.100.084002

[8] A.A. Coley, R.J. van den Hoogen, D.D. McNutt, Symmetric teleparallel geometries. Class. Quantum Gravity **39**, 22LT01 (2022). arXiv:2205.10719 [gr-qc] 10.1088/1361-6382/ac994a

[9] A.A. Coley, R.J. van den Hoogen, A. Landry, D.D. McNutt, Preprint: Spherically symmetric teleparallel geometries (in preparation) 10.1140/epjc/s10052-024-12629-5 PMC11266307 39049894

[10] S. Bahamonde, K.F. Dialektopoulos, C. Escamilla-Rivera, G. Farrugia, V. Gakis, M. Hendry, M. Hohmann, J.L. Said, J. Mifsud, E. Di Valentino, Teleparallel gravity: from theory to cosmology. Rep. Prog. Phys. **86**, 026901 (2023). arXiv:2106.13793 [gr-qc] 10.1088/1361-6633/ac9cef 36279849

[11] Y.-F. Cai, S. Capozziello, M. De Laurentis, E.N. Saridakis, teleparallel gravity and cosmology. Rep. Prog. Phys. **79**, 106901 (2016). arXiv:1511.07586 [gr-qc] 27599606 10.1088/0034-4885/79/10/106901

[12] C.G. Bohmer, E. Jensko, R. Lazkoz, Cosmological dynamical systems in modified gravity. Eur. Phys. J. C **82**(6), 500 (2022). arXiv:2201.09588 [gr-qc] 10.1140/epjc/s10052-022-10412-y

[13] E.N. Saridakis, Solving both and tensions in gravity. (2023). arXiv:2301.06881 [gr-qc]

[14] A.L. Besse, *Einstein Manifolds* (Springer Verlag, Berlin, 1987)

[15] S. Kobayashi, K. Nomizu, *Foundations of Differential Geometry and its Applications*, vol. 1 (Interscience, New York, 1963)

[16] G.S. Hall, *Symmetries and Curvature Structure in General Relativity* (World Science, Singapore, 2004)

[17] J. Brannlund, A.A. Coley, S. Hervik, Supersymmetry, holonomy and Kundt spacetimes. Class. Quantum Gravity **25**, 195007 (2008). arXiv:0807.4542 [gr-qc] 10.1088/0264-9381/25/19/195007

[18] H. Wu, Pac. J. Math. **20**, 351 (1967) 10.2140/pjm.1967.20.351

[19] H. Wu, Ill. J. Math. **8**, 291 (1964)

